# A Review on Hematuria in Children

**DOI:** 10.1100/tsw.2006.59

**Published:** 2006-03-08

**Authors:** Sarel Halachmi, David Kakiashvili, Shimon Meretyk

**Affiliations:** Department of Urology, Rambam Medical Center, Faculty of Medicine, Technion-Israel Institute of Technology, POB 9602, Haifa, Israel

**Keywords:** hematuria, children, evaluation, management, Israel

## Abstract

Asymptomatic hematuria is very common in pediatric and adolescent patients. In contrast to painless hematuria in adults, the differential diagnosis and investigative modalities in the pediatric population is vastly different. This article presents the major diseases that may cause hematuria in children and suggests an evaluation algorithm for the pediatric urologist.

## INTRODUCTION

Painless hematuria in adults is usually related (in more than 90% of cases) to a malignant process of the urinary tract, while hematuria in children, which is not uncommon (5–30% of asymptomatic children[1]), is related to a wide spectrum of conditions mostly not related to malignancy. Our paper presents a brief review of the conditions that may cause hematuria in children; diagnostic algorithms and management are discussed.

## ETIOLOGY OF HEMATURIA IN CHILDREN

- Trauma
- Congenital anomalies
- Infectious diseases and inflammatory processes
- Malignant tumors
- Metabolic disorders
- Systemic and autoimmune disorders
- Bleeding and blood cell disorders
- Vascular disorders

### Trauma

Urinary tract trauma is common in children and even minor trauma may cause extensive damage. The urinary tract system in children is less protected and, hence, more vulnerable to trauma. The muscles of the torso are less developed, the rib cage and the spine are softer, and the perirenal fat tissue is thinner[2].

The kidney of a child is relatively larger compared to the body dimensions and a larger portion of it is not protected by the rib cage. The paucity of protective renal tissue allows hypermobility of the kidney and deceleration trauma, such as falling and hyperextension, may induce severe tension on the renal pedicle, causing vascular damage, thrombosis, and even avulsion of the pedicle[3]. Congenital anomalies, such as obstruction and hydronephrosis, or shape anomalies make the anomalous kidney more vulnerable to trauma. For the same amount of force, such a kidney may suffer more damage than an adult kidney.

Lower urinary tract trauma is related to bicycle riding and tree climbing. Direct falling on the groin and perineal area or blunt trauma to the suprapubic region may result in bladder, urethra, and genital trauma. Children have a tendency to postpone urination during playing and a blunt injury to the lower abdomen may increase intravesicle pressure, leading to bladder rupture.

Hematuria in pediatric trauma is common and appears in 90–100% of injured children. The amount of hematuria is not directly related to the severity of the injury. Indications for diagnostic imaging in pediatric trauma are wider than in adults, and computerized tomography (CT) is the modality of choice[3,4].

## Congenital Anomalies

Hematuria as a presenting sign of urinary tract anomaly is extremely rare. Several case reports describing this phenomenon exist; however, in the era of prenatal ultrasonography, urological anomalies are easily diagnosed *in utero* and the problem managed immediately after birth. Obstructive lesions, such as ureteropelvic junction obstruction, may appear with urinary stones and hematuria; however, it is not clear yet whether the obstruction is the cause of stones or if there is coexistence of both a uromechanical disorder and a stone disorder.

## Infectious Diseases and Inflammatory Processes

Urinary tract infection may lead to hematuria. Symptomatology is related to the age of the child. Classical symptoms, such as dysuria, urgency, and frequency, usually appear in adolescent and older children; however, infants may have general symptoms such as fever, apathy or irritability, decreased appetite, and vomiting. School-age patients may describe lower abdominal pain and incontinence.

Urinalysis demonstrates hematuria, but in addition, white blood cells, nitrites, leukocyte esterase, and even bacteria are found. As vesicoureteral reflux is related to bacterial urinary tract infection in a child, further investigations, including voiding cystography, are indicated.

Hemorrhagic cystitis may appear a few days to months following chemotherapy and bone marrow transplantation[5]. Diagnosis in this case is based on the patient's history of recent treatment. Management is based on hydration and restoration of platelet count to increase clotting capability.

## Genitourinary Tumors (Wilms' Tumor)

Wilms' tumor is the most common urological malignancy in children. Abdominal pain, hematuria, and abdominal mass are the classic signs and symptoms. Diagnosis is made according to ultrasound and CT examinations. Treatment is based on the combined approach of a multidisciplinary team and includes chemotherapy and surgery; radiation therapy might be needed in advanced cases[8]. Several syndromes, such as hemihypertrophy, are related to the increased rate of Wilms' tumor in those patients. It is important to identify high-risk patients and follow them with serial ultrasounds for early detection of the malignant process.

## Genitourinary Tumors (Genitourinary Rhabdomyosarcoma)

This is the third most common malignant tumor in children following Wilms' tumor and neuroblastoma. More than half of new cases appear by the age of 5 years. Symptoms and signs include hematuria, dysuria, and urinary retention. Physical examination may reveal a hard mass in the area of the prostate, while in females, urethral or vaginal lesions may protrude beyond the orifice or the meatus, like a bunch of grapes. Biopsy should be taken and staging of the disease is done with CT or magnetic resonance imaging (MRI). Treatment is based on a combined approach of chemotherapy and surgery.

## Genitourinary Tumors (Urothelial Lesions)

- **Transitional cell carcinoma (TCC)**: This is a very rare malignant tumor in children and is rarely seen before the age of 14 years. Management of TCC in children is identical to adults, mandating endoscopic resection of the tumor and intravenous pyelography (IVP) to rule out the existence of lesions in the upper tract. Most tumors in young patients are superficial and well differentiated, but there is a high tendency (70%) of recurrence, as with all TCC lesions[10]. Urothelial carcinoma could appear as a secondary malignancy following chemotherapy, especially with cyclophosphamide[11], and adenocarcinoma of the bladder may appear in bladder exstrophy patients. These tumors usually appear 10–20 years following the genetic insult and usually not in childhood.
- **Bladder hemangioma**: This is a benign lesion composed of abnormal blood vessels in the bladder. Due to the improper structure of the vessels, painless hematuria may occur. Diagnosis can be made with Doppler ultrasound demonstrating a hypervascular structure in the bladder wall. The final diagnosis will be made endoscopicaly, and care should be taken not to biopsy the lesion, an act that could be complicated with severe bleeding. Asymptomatic lesions should be followed and bleeding lesions should be coagulated, preferably using laser energy[12].

## Metabolic Disorders (Urinary Stone Disease)

Urinary stones are less frequent in children than in adults (1:5,000 vs. 1:100). Urinary calculi in children are usually a consequence of congenital metabolic disorders such as cystinuria, hyperoxaluria, hypeuricosuria, and renal tubular acidosis. Nutritional disorders, including low-protein, high-carbohydrate diets, may induce calcium oxalate stones[13].

Signs and symptoms in children having urinary stones are irritability, abdominal pain, renal colic, hematuria, irritative urinary symptoms, and urinary tract infection. In every child with urinary calculi, the family history is crucial, as the existence of stones in other family members may be indicative of an inherited disorder. Congenital stone disease may affect younger children and cause early and severe renal damage.

In addition to metabolic evaluation imaging of the urinary tract, ultrasound, plain abdominal film, and IVP are all excellent modalities; however, recent studies have proven that nonenhanced spiral CT is the most accurate imaging method for urinary stones. Treatment of urinary stones in children is the same as in adults. Children have better chances than adults for spontaneous stone expulsion. Moreover, studies report higher success rates in children with shock wave lithotripsy (SWL), even in large stones. During SWL treatment, a protective lung shield should be applied to prevent shock wave lung contusions. Under these conditions, SWL in children is safe and highly effective[14,15].

## Systemic and Autoimmune Disorders

In most nonsurgical disorders that may cause hematuria, diagnosis and treatment are beyond the skill of the urologist.

- **Postinfectious pyelonephritis**: Caused by immune damage to the glumeruli postbacterial infection. Symptoms and signs, such as dark urine and edema, appear 10–21 days following streptococcal throat or skin infection. Urinalysis shows hematuria, casts, and proteinuria. Treatment consists of blood pressure control, fluid and electrolyte stabilization, and long-term follow-up of renal function[16].
- **IgA nephropathy**: Related to an accumulation of IgA immunoglobulin in the renal tissue. Acute upper respiratory or gastrointestinal disease will induce hematuria. Urinalysis shows red blood cells and protein. Treatment is based on immunosuppressive drugs and blood pressure control[17].
- **Alport syndrome**: Congenital x-linked dominant inherited nephritis leads to abnormal collagen synthesis causing a functional defect in the glumerular basement membrane and the inner ear. Patients suffer from deafness, hematuria, and proteinuria. There is no specific treatment for the disease[18].
- **Systemic lupus erythematosus**: An autoimmune disease causing multisystemic inflammatory phenomena. Renal damage includes hematuria, proteinuria, and renal failure. Treatment is based on immunosuppressive drugs and blood pressure control[19].
- **Benign familial hematuria**: A benign congenital autosomal dominant inherited disease. The disease presents with recurrent asymptomatic hematuria, while physical examination, laboratory tests, and urinary tract imaging are all normal. Diagnosis is based on the fact that hematuria is found in other family members, and by ruling out other diseases[20].

## Bleeding and Blood Cell disorders

Every bleeding disorder may present with hematuria. As most of these disorders are inherited, family history is easily obtained. Treatment is based on an accurate diagnosis of the disorder and bleeding function corrected accordingly[21,22,23].

- **Sickle cell anemia**: Hematuria is one of the most common signs in this disorder. Red blood cell sickling may obliterate small renal blood vessels leading to ischemia and hematuria. The ischemic process is painful and treatment includes pain control, even with narcotic drugs; hydration, body temperature maintenance, correction of metabolic acidosis, and blood oxygen saturation are all directed at stopping the sickling. In general, preventive measures such as proper liquid intake and avoidance of exposure to cold are recommended[24].

## Vascular Disorders

- **Hemolytic uremic syndrome**: A small, including renal, blood vessel disease. Patients may present with acute renal failure, anemia, hypertension, and neurological signs. Urinalysis shows hematuria and proteinuria. Maintaining platelet and red blood cell counts and dialysis are the main treatment options[25].
- **Henoch Schoenlein purpura**: Small blood vessel vasculitis presenting with purpuric skin lesions, abdominal and joint pain, hematuria, and proteinuria. As many as 50% of patients may develop end-stage renal failure. Treatment is based on steroids and pain control[26].

## DIAGNOSTIC ALGORITHM AND MANAGEMENT

### Medical History

Information on trauma is usually available. It is important to emphasize that, in children, even minor trauma can result in extensive genitourinary tract damage. In adult patients, classic symptoms of urinary tract or upper respiratory tract illness can be obtained. Time between onset of symptoms to the appearance of hematuria can provide a clue about the nature of the disease. Concomitant bleeding from other organs may indicate coagulation disorders; a family history of bleeding disorders, renal, hearing, and sickle cell disease should be obtained.

### Physical Examination

Signs of blunt or penetrating trauma should be located, when there is a history of trauma. Genitalia should always be examined and, in cases of trauma, signs of contusion, hematoma, or lacerations should be identified. Lesions of the genitalia without a clear history of trauma should raise the suspicion of child abuse. When a genitourinary mass, such as rhabdomyosarcoma, is part of the differential diagnosis, a rectal examination should be performed also. Examination of the abdomen demonstrating tenderness of the flank could lead to the diagnosis of a urinary tract infection or stone disease; abdominal, retroperitoneal, and pelvic masses are relatively easier to palpate in young children than in adults as the abdominal wall layers are less developed.

### Laboratory Tests

Urinalysis should verify the existence of red blood cells. The concomitant presence of red blood cells, proteins, nitrites, and positive leucocytes esterase assay is pathognomonic for urinary tract infection; urine culture will provide the final diagnosis. Urinary casts and protein will direct us toward the diagnosis of renal glomerular and parenchymatic diseases. Urine microscopy can also identify crystals that may appear in stone disease. Red blood cell morphology can also contribute to the identification of the source of bleeding, as dysmorphic red blood cells indicate glomerular bleeding and normal morphology is usually attributed to urothelial bleeding[27]. As many of the disorders that cause hematuria affect renal function, serum urea, creatinine, and electrolytes should be tested.

### Evaluation and Management

Trauma patients should be managed according to their hemodynamic status and concomitant injuries. In stable children with trauma, the gold standard for imaging is CT. If urethral or bladder trauma is suspected, urethrocystography should be performed. Abdominal masses should be evaluated with ultrasound to differentiate between cystic/hydronephrotic masses and solid masses. Hydronephrosis will be evaluated by the urologist with a cystogram and renal scan. Solid lesions are considered malignant and extensive work-up including CT and MRI will be performed. In cases of urinary tract infection, a cystogram is recommended because of the high rate of vesicoureteral reflux (VUR) in these patients. Prophylactic antibiotics should be given until VUR is ruled out. Urinary stone disease should be managed by the urologist and nephrologist together. In many cases, a childhood urinary stone is due to inherited disease; hence, a complete metabolic evaluation should be conducted by the nephrologist. “Mechanical” evaluation will be done by the urologist. CT and IVP are excellent modalities to identify and plan treatment for urinary stones; however it should be kept in mind that these children will need many diagnostic imaging tests and will have extensive exposure to radiation; therefore, ultrasound is a relatively good modality for follow up measures.

A history of hematuria following upper respiratory or gastrointestinal disease, in addition to signs of edema, hypertension, and rash, should direct us towards the diagnosis of glomerular diseases. If the urologist is the primary physician, he can promote the diagnosis by taking throat cultures, complement levels, and antistreptolysin. Care should be taken to measure the patient's blood pressure and to verify that renal function is normal. Management should be under the supervision of a pediatrician; however, if hypertension, edema, or impaired renal function exists, the patient should be cared for in a hospital facility.

## CONCLUSION

Hematuria in children has a wide differential diagnosis, mandating different approaches and management compared to adult patients. Family history, concomitant symptoms and signs, physical examination, urinalysis, and blood tests may direct us towards an accurate diagnosis. Most of the disorders that may cause hematuria are "nonsurgical, nonurological" diseases and should be managed by a pediatrician. In a minority of cases, invasive diagnostic procedures will need to be performed. Primary diagnostic imaging using ultrasound is widely used.

## BIOSKETCHES

**Sarel Halachmi**, Pediatric Urology Service, Department of Urology, Rambam Medical Center and Faculty of Medicine, Technion-Israel Institute of Technology, POB 9602, Haifa, Israel. E-mail: s_halachmi@rambam.health.gov.il

**David Kakiashvili**, Department of Urology, Rambam Medical Center and Faculty of Medicine, Technion-Israel Institute of Technology, POB 9602, Haifa, Israel.

**Shimon Meretyk**, Department of Urology, Rambam Medical Center and Faculty of Medicine, Technion-Israel Institute of Technology, POB 9602, Haifa, Israel.
